# Predictability of Postural and Standing Balance from a Clinical Impairment Battery in Children with Cerebral Palsy: A Cross-Validated Regression and Hurdle Model Analysis

**DOI:** 10.3390/bioengineering13101137

**Published:** 2026-09-28

**Authors:** Ahmed Fekry Salman, Jamal I. Al-Nabulsi, Ali Olamat, Faten H. Abdelaziem, Hoda Abd E. A. El-Talawy, Ahmed E. Fayed

**Affiliations:** 1 Physical Therapy Department, Faculty of Allied Medical Sciences, Al-Ahliyya Amman University, Amman 19111, Jordan; 2 Department of Medical Engineering, Faculty of Engineering, Al-Ahliyya Amman University, Amman 19111, Jordan; j.nabulsi@ammanu.edu.jo (J.I.A.-N.); a.olamat@ammanu.edu.jo (A.O.); 3 Department of Physical Therapy for Pediatrics, Faculty of Physical Therapy, Cairo University, Cairo 12613, Egypt; faten.hassan@pt.cu.edu.eg (F.H.A.); hoda_eltalawy@hotmail.com (H.A.E.A.E.-T.); 4 Faculty of Physical Therapy, October 6 University, Giza 12573, Egypt; 5 Physical Therapist at National Institute of Neuromotor System, Cairo 12613, Egypt; drahmedfayed88@gmail.com

**Keywords:** cerebral palsy, postural balance, machine learning, regression analysis, muscle strength, child, preschool, motor skills

## Abstract

(1) Background: Balance determines functional status in cerebral palsy (CP), yet its predictability from clinical impairment measures remains unclear. This study aims to evaluate the predictability of three balance targets using a clinical impairment battery and determine whether standing balance comprises discrete and graded components. (2) Methods: A total of 173 children with CP (aged 0.9–9.0 years) were assessed using the Early Clinical Assessment of Balance (ECAB) (ECAB total, Part 1: head/trunk control, Part 2: standing). Spasticity, muscle strength, posture, tone asymmetry, age, sex, and CP subtype were analyzed as predictors using cross-validated regression models and a two-part hurdle model. (3) Results: Head and trunk control demonstrated high predictability (R^2^ = 0.807), while the ECAB total score achieved R^2^ = 0.671. Standing balance showed moderate predictability in standard regression (R^2^ = 0.519). The hurdle model decomposed this: it discriminated standing capability with high accuracy (AUC = 0.982) but explained only 41.4% of graded variance among standers (R^2^ = 0.414), clarifying rather than improving overall prediction. Muscle strength was the primary predictor across all models. (4) Conclusions: Within this cohort, clinical impairments reconstruct head and trunk control and predict whether a child can stand but do not explain standing quality; external validation is still required. The ECAB standing subscale captures graded information not reconstructable from the impairment battery as specified (which by design excludes global severity classification) and should be measured directly.

## 1. Introduction

Balance—the maintenance of the body’s center of mass over its base of support—is among the strongest determinants of independent function in children with cerebral palsy (CP) [1,2]. Impaired postural control limits sitting for play and feeding, and impaired standing balance limits ambulation; both are primary targets of physiotherapy. Balance is not a single construct. Proximal postural control, expressed as head and trunk stability, develops earlier and provides the foundation on which distal standing balance is built, and the two can dissociate: a child may sit securely yet be unable to stand unsupported [3]. Clinical assessment reflects this structure. The Early Clinical Assessment of Balance (ECAB), developed for young children with CP, separates head and trunk control (Part 1) from standing balance (Part 2) and has established reliability and construct validity against the Gross Motor Function Measure [4,5].

Alongside balance, pediatric CP assessment routinely quantifies spasticity with the Modified Ashworth Scale (MAS) [6], functional strength with the Functional Strength Assessment (FSA) [7,8], and posture and flexibility with the Spinal Alignment and Range of Motion Measure (SAROMM) [9,10]. These impairment measures are correlated with balance, but the extent to which they determine it has not been quantified in a predictive framework, and it is not known whether the postural and standing components of balance are predictable to the same degree. If they are not, we hypothesize that the component least predictable from impairment measures may be the one most in need of direct measurement—a working hypothesis rather than a claim of intrinsic unpredictability.

A specific statistical feature of standing balance has not been addressed in previous predictive work. In a young CP cohort a substantial fraction of children cannot stand at all and therefore score zero on the standing subscale, producing a distribution with a spike at zero rather than a continuous spread. A single regression fitted to such a distribution conflates two questions—whether a child can stand and how well a standing child balances—and its overall fit is difficult to interpret. A two-part, or hurdle, model separates these questions and is the appropriate design when an outcome has a floor [11]. In the ECAB, the standing items are scored by the child’s ability to maintain standing with progressively less support, so a Part 2 score of zero corresponds to a child who cannot maintain standing under any of the graded conditions (per the ECAB scoring definition).

This study quantified the predictability of balance from a routinely administered impairment battery in 173 children with CP, using a leakage-controlled cross-validation protocol, and tested whether standing balance decomposes into separable discrete and graded components. The contributions are: (1) a comparison of the predictability of the ECAB total and its two parts under identical predictors and validation; (2) a demonstration that nine regression families converge on the same ceiling for standing balance, distinguishing an informational limit from a modeling limit; (3) a hurdle model decomposition separating the ability to stand from the graded quality of standing; and (4) an interpretability analysis identifying the impairment measures that drive each balance component.

## 2. Materials and Methods

**Figure 1:**
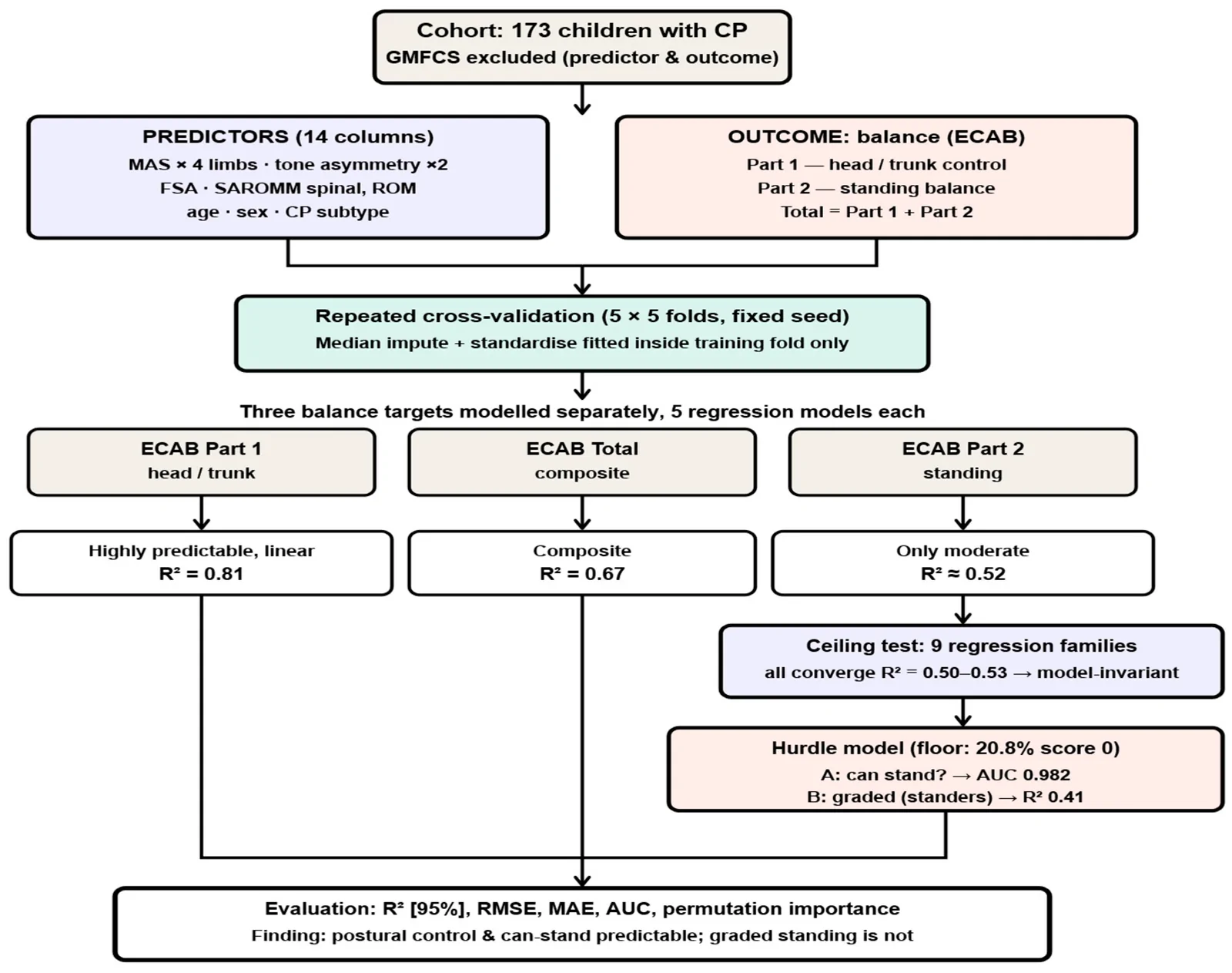
Analysis pipeline overview. A battery of fourteen predictors (spasticity, tone, strength, asymmetry, and posture, demographics, and CP type) predicted three ECAB balance tasks through repeated cross-validation with fold-specific data preprocessing. Head and trunk control proved very predictive using a linear model, whereas standing was moderately predictive, requiring a nine-model ceiling test and a hurdle model to split standing ability (AUC 0.982) from standing quality (R^2^ 0.41).

The analysis involved four steps that can be seen in Figure 1 below. Balance was estimated using the ECAB and predicted from a fourteen-column impairment battery, using a leakage-controlled cross-validation process, through which the Gross Motor Function Classification System [12,13] was excluded, both because its levels are partly defined by standing and walking ability (making it partly circular as a predictor of standing balance) and because the predictability of the classification itself from this battery is examined in a companion study. There were three targets in balance, which included head/trunk control, standing balance, and the total of the two. Each target was modeled using five different regression models. Since standing balance had very poor prediction, it was subjected to further tests using nine different regression models to differentiate between information limit and modeling limit. The floor value of standing balance was set at zero, and therefore a two-stage hurdle model was used, whereby the standing is separated from the quality of standing.

### 2.1. Participants and Cohort Overlap

A cross-sectional dataset of 173 children with CP was analyzed (94 male, 79 females; age 0.9–9.0 years, mean 3.2, standard deviation 1.1; median 3.0, IQR 2.3–3.9; the age distribution is right-skewed, so median and IQR are the more representative summary). Records were obtained from the outpatient clinic at the Faculty of Physical Therapy, Cairo University, Egypt, from September 2024 to May 2025. This study was approved by the ethical review committee at Cairo University, Faculty of Physical Therapy (Approval Number: P.T.REC/012/005209). The study protocol was registered prospectively with ClinicalTrials.gov under the number NCT06601569, and a parent or guardian of each child gave written informed consent. The composition of the cohort and the distribution of the three balance outcomes are summarized in Table 1. Standing balance (ECAB Part 2) was zero in 36 children (20.8%), who could not stand, producing the floor that motivated the hurdle model of Section 2.5; head and trunk control (Part 1) had no floor. The battery was administered by [2] trained pediatric physiotherapists. To maintain the methodological rigor, the same clinician scored the child’s impairment battery and balance outcome, while assessors remained blinded to all other clinical scores and prior test results. All evaluations were conducted in a standardized sequence and remain constant across participants. Any potential shred-rater dependency introduced by this protocol is addressed in Section 4.3.

### 2.2. Outcome and Predictor Definition

The outcome was balance, operationalized as the ECAB. Three targets were modeled separately: the ECAB total, Part 1 (head and trunk control) and Part 2 (standing balance). Because the total is the exact sum of the two parts, the parts were never used to predict the total or one another. Predictors were age; sex; the four MAS segment scores (right and left, upper and lower limb), recoded from the ordinal grades to consecutive integers; two engineered tone asymmetry features, defined as the absolute right–left difference in the upper and in the lower limb; the FSA total; the SAROMM spinal alignment and range-of-motion subscores; and topographical CP subtype entered as indicator variables. The predictor set contained fourteen columns and excluded the ECAB entirely. The fourteen columns were: age; sex; the four MAS segment scores; the two MAS tone asymmetry features; the FSA total; the SAROMM spinal-alignment and range-of-motion subscores; and three CP-subtype indicators (hemiplegia, quadriplegia, other (ataxia and dyskinesia)), with diplegia as a reference. The FSA total summarizes functional strength across antigravity movements (including sit-to-stand and limb-raising against resistance). The two tone asymmetry features are absolute differences of the MAS segment scores and are algebraically dependent on them.

### 2.3. Preprocessing and Leakage Control

Both preprocessing operations—median imputation of any missing values, followed by standardization to zero mean and unit variance—were fitted on the training partition of each cross-validation fold and applied unchanged to the held-out partition, so that no information from held-out children influenced the transformation of the training data. No feature selection or outlier removal was performed on pooled data, and model hyperparameters were fixed a priori rather than tuned, removing selection as a channel for optimistic bias [14,15]. Missing data were minimal (FSA 1.7%, ECAB total 0.6%; all other predictors complete), so fold-internal median imputation is unlikely to have biased the fitted relationships. Categorical CP subtype was encoded as fixed indicator variables (diplegia as a reference) and required no fold-internal fitting.

### 2.4. Regression Models

Five regression models spanning distinct inductive biases were compared for each target: ordinary least squares; elastic net regression with the mixing and penalty parameters selected by inner cross-validation [16]; a random forest of 300 trees with a minimum leaf size of five; gradient-boosted trees with 200 iterations, depth three and learning rate 0.1 [17,18]; and support vector regression with a radial basis kernel. To test whether the predictability of standing balance was limited by model capacity or by the information in the predictors, an extended comparison for Part 2 retained the three non-linear primary models (random forest, gradient boosting and support vector regression) and added extremely randomized trees, histogram gradient boosting, kernel ridge regression, k-nearest-neighbors regression, a multilayer perceptron and a Gaussian process regressor, giving nine regression families in total [19]; all models were implemented in scikit-learn [20]. Full model implementations, hyperparameters, software (scikit-learn 1.9.0) version and the random seed are listed in Table A1 (Appendix A).

### 2.5. Hurdle Model for Standing Balance

The standing subscale was zero for 36 of 173 children (20.8%), who could not stand, producing a semicontinuous distribution with a floor at zero. A two-part hurdle model was therefore specified. Part A modeled the probability of any standing ability as a binary classification of Part 2 greater than zero, using logistic regression and a random forest with inverse-frequency class weighting. Part B modeled the graded standing score among the 137 children with a positive Part 2, using the five regression models of Section 2.4. Let s denote the standing score, x the predictor vector, and 1(·) the indicator function; the hurdle prediction combines the two parts as

$$\hat{s}\left(x\right)=P\hat{r}\left(s>0|x\right)>0.5\;\;\;\hat{g}\left(x|s>0\right):0$$

where $\hat{g}$ is the Part B regression fitted on standers only. Parts A and B were each evaluated by cross-validation, and the combined prediction was evaluated on the full sample for comparison with the direct regression.

### 2.6. Validation and Evaluation

All models were evaluated by repeated cross-validation with five folds and five repeats for regression targets and ten repeats for the binary Part A, with a fixed partition seed so that all models were compared on identical partitions. Regression performance was summarized by the coefficient of determination R^2^, the root-mean-square error (RMSE) and the mean absolute error (MAE), each computed on the concatenated out-of-fold predictions of a repeat; the repeat-level values gave central estimates and 2.5th–97.5th-percentile intervals. R^2^ was reported relative to an intercept-only baseline. The binary Part A was summarized by accuracy, balanced accuracy, and the area under the receiver operating characteristic curve. Predictor contributions were quantified by permutation importance over 40 permutations, expressed as the decrease in R^2^ (regression) or balanced accuracy (Part A) when a predictor was permuted [21]. Because the same children recur across folds and repeats, these out-of-fold predictions are not independent, so the intervals reflect fold partition variability and understate true generalization uncertainty [22]. Model comparisons were assessed with a corrected resampled paired t-test rather than by interval overlap, and the per-target choice of a best model among five (or nine) is a selection procedure, not a confirmatory test; intervals are not adjusted for multiplicity.

## 3. Results

### 3.1. Predictability of the Three Balance Targets

**Figure 2:**
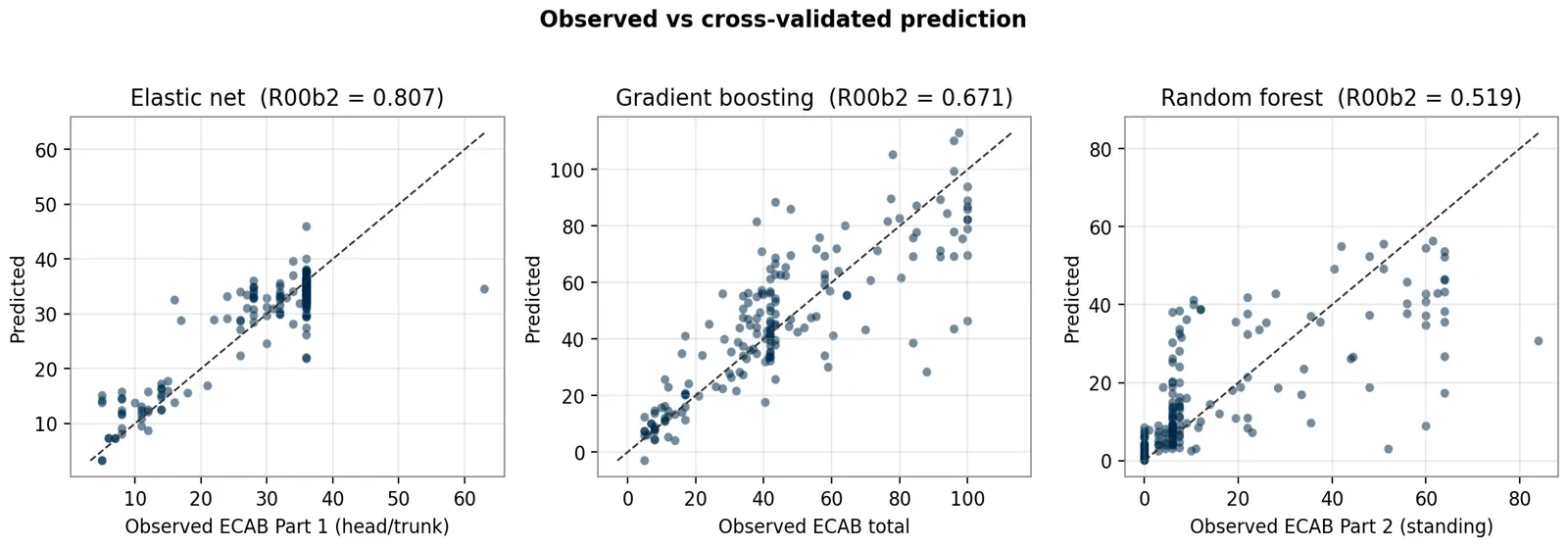
Observed against cross-validated predicted values for the three balance targets: head and trunk control (ECAB Part 1), ECAB total, and standing balance (ECAB Part 2). The identity line is shown. The floor of standing scores at zero is visible in the right panel.

Table 2 reports cross-validated performance for the three balance targets under the five primary regression models. Head and trunk control (Part 1) was the most predictable target, reaching R^2^ = 0.807 with elastic net regression; for this target the two linear models outperformed all three non-linear ensembles, indicating a predominantly additive relationship with the impairment predictors. The ECAB total was moderately predictable, with gradient boosting and the random forest reaching R^2^ = 0.671 and 0.670, respectively. Standing balance (Part 2) was the least predictable, with the best of the five models reaching R^2^ = 0.519 and the non-linear models exceeding the linear ones by a small margin, indicating threshold rather than additive structure. Figure 2 shows observed against predicted values for each target.

### 3.2. A Model-Invariant Ceiling for Standing Balance

To determine whether the moderate predictability of standing balance reflected a limitation of the primary models or of the predictors, nine regression families were compared on Part 2 (Table 3). The estimates ranged narrowly from R^2^ = 0.438 (k-nearest neighbors) to R^2^ = 0.525 (kernel ridge), and the six tree-ensemble, kernel and Gaussian process methods all fell between 0.502 and 0.525, differences within their overlapping intervals. The multilayer perceptron failed (R^2^ = −0.159), consistent with an insufficient sample for a neural network. The appropriately regularized families (random forest, extra trees, histogram gradient boosting, kernel ridge and gradient boosting) converged at R^2^ ≈ 0.50–0.53, while higher-capacity and distance-based models (multilayer perceptron, Gaussian process, support vector regression) did not exceed them; at this sample size the latter is best read as a capacity–sample-size limitation rather than independent confirmation of a ceiling. No more flexible model recovered additional signal from these predictors at this sample size.

### 3.3. Hurdle Model Decomposition of Standing Balance

**Figure 3:**
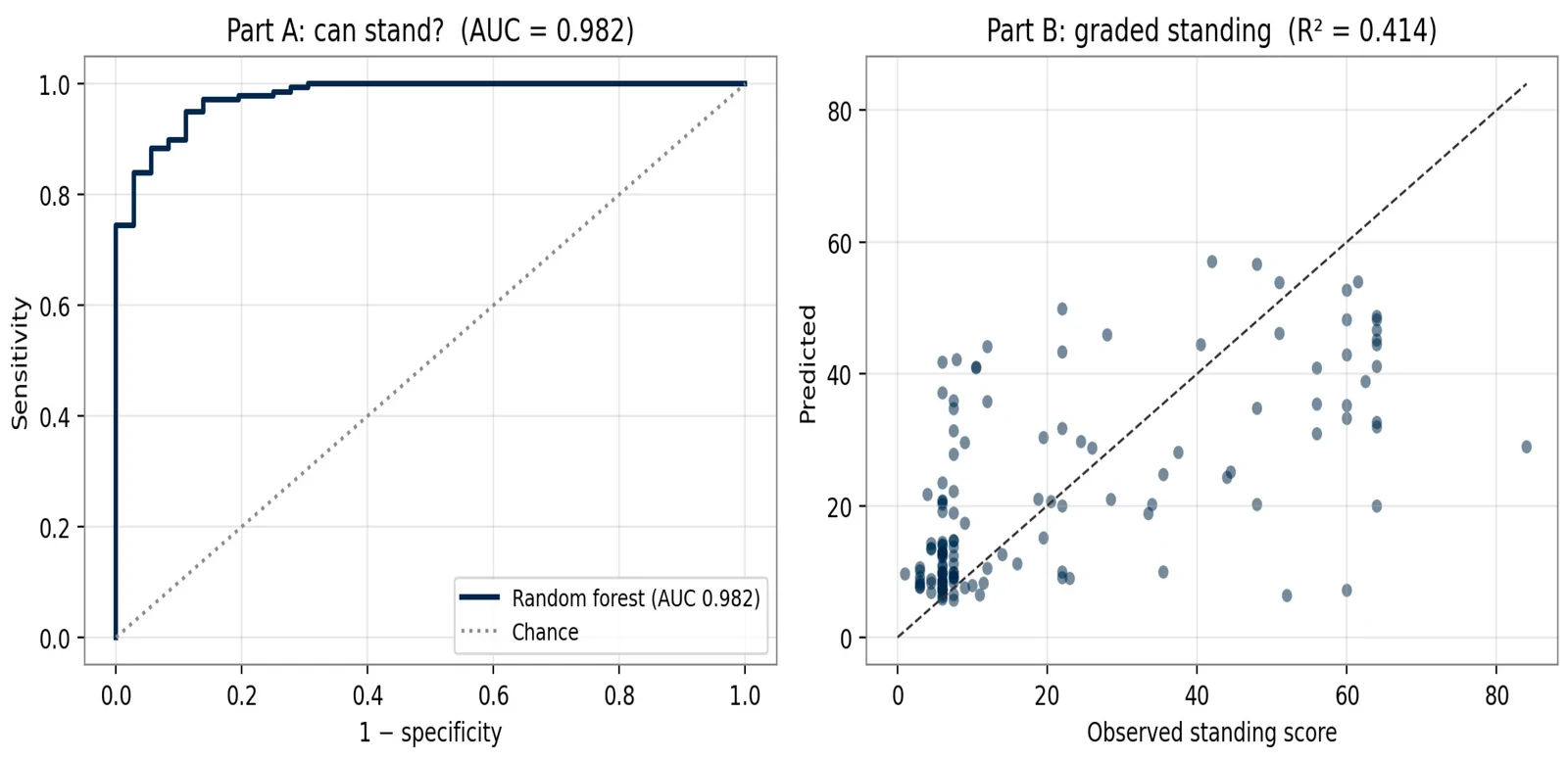
Hurdle model decomposition of standing balance. (**Left**): receiver operating characteristic curve for the ability to stand (Part A, random forest, AUC 0.982). (**Right**): observed against predicted graded standing score among standers (Part B, random forest, R^2^ 0.414).

The hurdle model separated the two questions conflated in the direct regression (Table 4). In Part A, the ability to stand at all was classified with high accuracy: the random forest reached an area under the curve of 0.982 (interval 0.976–0.984), balanced accuracy of 0.912 and accuracy of 0.938, and logistic regression reached an area under the curve of 0.968. In Part B, restricted to the 137 children who could stand, the graded standing score was only moderately predictable, with the random forest reaching R^2^ = 0.414 (interval 0.363–0.468) and no model exceeding it materially. The combined hurdle prediction reached R^2^ = 0.526 on the full sample, statistically indistinguishable from the direct random-forest value of 0.519 (corrected resampled paired t-test, non-significant). The decomposition therefore did not improve overall prediction, but showed that the moderate direct R^2^ combined a highly discriminable discrete component (high AUC under internal cross-validation, not determinism) with a weakly predictable graded component. Figure 3 shows the receiver operating characteristic curve for Part A and the observed-against-predicted plot for Part B.

### 3.4. Predictor Contributions

**Figure 4:**
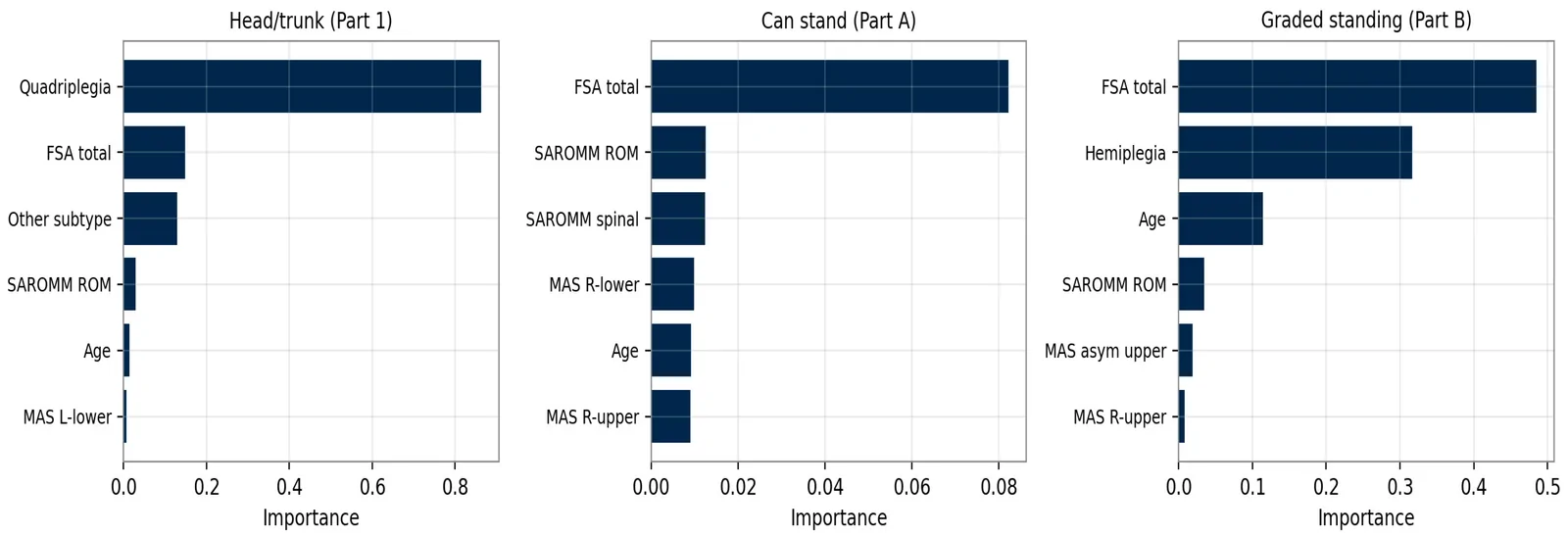
Permutation importance for the three balance models. Bars show the decrease in R^2^ (Part 1 and Part B) or balanced accuracy (Part A) when each predictor is permuted, over 40 permutations. Functional strength leads every target.

Permutation importance is reported in Table 5. Functional strength was the leading impairment predictor of every balance target, and in each case ranked above every individual spasticity measure. For head and trunk control, the quadriplegia indicator carried the largest importance, reflecting the near-constant low scores of that subtype, followed by the FSA. For the ability to stand (Part A), the FSA was the dominant predictor, ahead of the SAROMM subscores. For the graded quality of standing (Part B), the FSA and the hemiplegia indicator led, with age contributing further. Spasticity measures contributed modestly throughout, and tone asymmetry contributed little to balance despite its value for subtype classification. Figure 4 displays the three importance profiles. Because the asymmetry features are algebraically linked to the MAS scores, permutation importance may split credit across dependent predictors; and subtype indicator importance rests on small subgroups (quadriplegia *n* = 31, other *n* = 10, GMFCS V *n* = 4) and is unstable—removing the 14 children in GMFCS V and the “other” subtype shifted the quadriplegia importance from 0.86 to 1.21. The FSA was strongly correlated with the standing outcome before modeling (Spearman ρ = 0.76 overall, 0.48 among standers), so part of its weight may reflect overlap between two functional measures. These rankings are indicative rather than definitive.

## 4. Discussion

### 4.1. Principal Findings

Three principal findings arose from the analysis. First, the predictability of balance from an impairment battery varied greatly depending on which balance construct was modeled: head and trunk control was predicted very accurately and additively (R^2^ = 0.807, best linear model), while standing balance was predicted only moderately (R^2^ ≈ 0.52). Second, the predictability of standing balance appeared to reach an empirical ceiling, in that nine different regression families converged to R^2^ = 0.50–0.53, indicating that the unexplained variance is not reducible through more flexible modeling. Third, the hurdle model revealed that standing balance consists of a discriminable under internal cross-validation discrete aspect—whether a child is able to stand, measured by an area under the curve of 0.982—and a weakly predicted graded aspect—how well a standing child balances, R^2^ = 0.414.

The second finding consistently obtained was that the functional strength, not spasticity, was the leading predictor of every balance target variable, outranking every individual MAS score. This result is consistent with studies showing that muscle weakness is an independent impairment in CP, rather than a consequence of spasticity, and with arguments for strength training in this population [7,8,23,24].

### 4.2. Interpretation

The dissociation of the two balance components has a straightforward interpretation. Head and trunk control is a proximal postural skill closely linked to the measured impairments—strength of the trunk, spinal alignment and subtype—and, hence, is reconstructable from them. The ability to stand is a threshold that these impairments can predict accurately, as a child without the required strength and alignment cannot surpass it. In contrast, the graded quality of standing in children who are standing may depend on dynamic mechanisms of posture—such as anticipatory adjustment, sensory integration and reactive control—that were not measured in this study and are not captured by the static impairment battery; these mechanisms are proposed as hypotheses rather than demonstrated here, consistent with evidence that postural balance in other neurodevelopmental populations depends on sensory and cognitive factors beyond conventional impairment measures [25,26,27,28]. Therefore, the finding that the graded component was not well predicted by the battery in this cohort is not an indicator of inadequate coverage of measurements, but a reflection of what the constructs that impairment scores represent.

The practical implication is clear. As the battery predicts the ability to stand accurately but not the graded quality of standing, the ECAB standing subscale contains information that cannot be deduced from spasticity, strength and posture as measured in this battery (which excludes global severity classification) and should be measured separately. The hurdle structure also provides a more interpretable clinical prediction: a model could report the probability of a child being able to stand, and, for those who are standing, a graded prediction with its uncertainty.

### 4.3. Limitations

The ECAB is age-banded and the cohort covers a large age span, such that age only partly controls for this covariate. Balance was assessed by clinical ordinal instrument rather than posturography; thus, the residual variability on graded standing can no longer be interpreted mechanistically in terms of postural control from these data. Hyperparameters were chosen a priori, which precludes optimistic reporting but leaves open whether different results would emerge using optimized parameters, unlikely in light of the convergence of nine different model families. Finally, the MAS includes both neurologic and non-neurologic resistance and was recoded into equal intervals—this is an assumption unlikely to be true. As with SAROMM, recoding the ordinal MAS to consecutive integers imposes an equal-interval assumption the scale does not satisfy, notably the irregular step between grades 1 and 1+; this affects the linear, kernel ridge, k-nearest-neighbor and Gaussian process models in particular. All children came from a single Cairo University outpatient clinic over eight months; referral to one tertiary center can skew severity (GMFCS V, *n* = 4; level III, 45.7%), so the finding should be read as holding within this referral population until externally validated. If one clinician scored both a child’s impairment battery and balance outcome in a single session, a shared-rater dependency could inflate the association. Entering age as a cubic spline rather than a linear term did not improve standing prediction (random forest ΔR^2^ = +0.001), indicating no materially recoverable age non-linearity. The design is cross-sectional and every performance estimate derives from internal cross-validation rather than an external cohort; the GMFCS distribution is also unbalanced (level III *n* = 79 vs. level V *n* = 4), which may affect subgroup performance. External validation in an independent population is therefore a prerequisite for clinical use.

### 4.4. Future Work

Posturography, or inertial movement tracking while standing, will provide an answer to the question of whether a dynamic postural control mechanism explains the variability not accounted for by the battery. Longitudinal assessment is needed to investigate whether the stand/no-stand cut-off and graded standing score have distinct developmental profiles, a possibility compatible with but not demonstrated by the present cross-sectional dissociation. An external validation study in an independent population, including model calibration [29,30], is a prerequisite for the clinical implementation. The strength/balance relationship should be investigated, considering the consistent dominance of strength over spasticity in all balance tasks.

## 5. Conclusions

In CP patients, the ability to predict balance by the use of an impairment battery test was highly dependent on the balance domain. Control of the head and trunk position was highly predictable within the present dataset using a linear model (R^2^ = 0.807; ~19% of variance unexplained), whereas whether a child could stand was classified with high accuracy (area under the curve 0.982) under internal cross-validation. However, the graded quality of standing was predicted only poorly, and nine regression models converged to a similar level, with more flexible models providing no gain at this sample size. Functional strength, rather than spasticity, was the predominant predictor of standing balance through all regression procedures. Hence, standing balance is a combination of a discrete domain, which can be measured by an impairment measure test, and of a graded one that the present battery did not predict well in this cohort.

## Figures and Tables

**Table 1:** Composition of the analyzed cohort and distribution of the balance outcomes (*n* = 173). ECAB = Early Clinical Assessment of Balance; SD = standard deviation.

Characteristic	Category	*n*	Percentage (%)
Sex	Male	94	54.3
	Female	79	45.7
Age (years)	Mean ± SD	3.2 ± 1.1	—
	Median [IQR]	3.0 [2.3–3.9]	—
	Range	0.9–9.0	—
GMFCS level	I	41	23.7
	II	14	8.1
	III	79	45.7
	IV	35	20.2
	V	4	2.3
CP subtype	Diplegia	89	51.4
	Hemiplegia	43	24.9
	Quadriplegia	31	17.9
	Other (ataxia, dyskinesia)	10	5.8
Balance outcome (ECAB)	ECAB Part 1 (head/trunk)	28.6 ± 10.8	median 36 (IQR 24–36)
	ECAB Part 2 (standing)	16.6 ± 20.9	median 6 (IQR 3–22); 36 (20.8%) = 0
	ECAB total	45.1 ± 27.1	median 42 (IQR 28–58)

**Table 2:** Cross-validated regression performance for the three balance targets (five primary models, 5 × 5-fold). Best model per target in the text. R^2^ intervals are 2.5th–97.5th percentiles over repeats.

Target	Model	R^2^	RMSE	MAE
ECAB Part 1 (head/trunk)	Elastic net	0.807	4.73	3.42
	Linear (OLS)	0.799	4.83	3.55
	Random forest	0.771	5.15	3.31
	Gradient boosting	0.752	5.56	3.54
	Support vector regression	0.483	7.87	5.31
ECAB total	Gradient boosting	0.671	15.88	11.26
	Random forest	0.670	15.91	11.64
	Linear (OLS)	0.647	16.45	12.35
	Elastic net	0.646	15.94	11.83
	Support vector regression	0.229	23.77	17.28
ECAB Part 2 (standing)	Random forest	0.519	14.47	9.54
	Gradient boosting	0.510	14.60	9.43
	Linear (OLS)	0.474	15.14	9.71
	Elastic net	0.450	15.15	11.03
	Support vector regression	−0.014	20.96	11.59

**Table 3:** Extended model comparison for standing balance (ECAB Part 2, *n* = 173, standard deviation 20.88). Nine regression families under identical predictors and validation. R^2^ intervals are 2.5th–97.5th percentiles.

Model	R^2^	95% Interval	RMSE	MAE
Kernel ridge (RBF)	0.525	0.508–0.558	14.38	9.99
Random forest	0.519	0.488–0.547	14.47	9.54
Extra trees	0.510	0.489–0.539	14.61	9.77
Gradient boosting	0.510	0.463–0.563	14.60	9.43
Histogram gradient boosting	0.502	0.481–0.542	14.73	10.17
Gaussian process	0.502	0.463–0.549	14.72	10.28
Support vector regression	0.474	0.443–0.537	15.14	9.71
k-nearest neighbors	0.438	0.421–0.455	15.66	10.10
Multilayer perceptron	−0.159	−0.282–−0.084	22.46	14.90

**Table 4:** Hurdle model decomposition of standing balance. Part A classifies the ability to stand (Part 2 > 0); Part B regresses the graded score among standers (*n* = 137). Combined prediction evaluated on the full sample.

Component	Model	Metric	Value	95% Interval
Part A: can stand?	Random forest	AUC	0.982	0.976–0.984
	Random forest	Balanced accuracy	0.912	—
	Logistic	AUC	0.968	0.963–0.976
Part B: graded (standers)	Random forest	R^2^	0.414	0.363–0.468
	Kernel ridge	R^2^	0.401	0.394–0.410
Combined hurdle	RF + RF	R^2^ (full sample)	0.526	0.496–0.550
Direct regression	Random forest	R^2^ (full sample)	0.519	0.488–0.547

**Table 5:** Permutation importance for the three balance models (random forest, 40 permutations). Values are the decrease in R^2^ (Part 1, Part B) or balanced accuracy (Part A) when a predictor is permuted. Predictors below 0.010 omitted.

Head/Trunk Control (Part 1)	Imp.	Can Stand (Part A)	Imp.	Graded Standing (Part B)	Imp.
Quadriplegia	0.889	FSA total	0.095	FSA total	0.447
FSA total	0.140	SAROMM spinal	0.016	Hemiplegia	0.331
Other subtype	0.127	SAROMM ROM	0.016	Age	0.121
SAROMM ROM	0.029	Age	0.012	SAROMM ROM	0.036
Age	0.015	MAS right upper	0.010	MAS asymmetry upper	0.018

## Data Availability

The dataset is available from the corresponding author on reasonable request subject to institutional approval. The analysis code reproducing all preprocessing, models, tables and figures is available from the corresponding author on reasonable request.

## Abbreviations

The following abbreviations are used in this manuscript:

CP	Cerebral palsy
ECAB	Early Clinical Assessment of Balance Scale
GMFCS	Gross Motor Function Classification System

## Appendix A. Model Implementations and Hyperparameters

**Table A1:** Implementation, hyperparameters and random seed for every model, under the identical leakage-controlled protocol (scikit-learn; fold-internal median imputation + standardization; Repeated K Fold 5 × 5 for regression, Repeated Stratified K Fold 5 × 10 for Part A) [R3-5/R3-13].

Model	Role	Scikit-Learn Class	Key Hyperparameters
Ordinary least squares	Primary	Linear Regression	fit_intercept = True; no tuning parameters
Elastic net	Primary	Elastic Net CV	l1_ratio ∈ {0.1,0.5,0.7,0.9,0.95,0.99,1}; α by 5-fold inner CV (100-α path); max_iter = 5000
Random forest	Primary	Random Forest Regressor	n_estimators = 300; min_samples_leaf = 5; max_features = 1.0; bootstrap = True
Gradient boosting	Primary	Gradient Boosting Regressor	n_estimators = 200; max_depth = 3; learning_rate = 0.1; subsample = 1.0
Support vector regression	Primary	SVR	kernel = “rbf”; C = 1.0; epsilon = 0.1; gamma = “scale”
Extra trees	Extended	Extra Trees Regressor	n_estimators = 300; min_samples_leaf = 5; bootstrap = False
Histogram gradient boosting	Extended	Hist Gradient Boosting Regressor	learning_rate = 0.1; max_iter = 100; max_leaf_nodes = 31; min_samples_leaf = 20; l2_regularization = 0
Kernel ridge	Extended	Kernel Ridge	kernel = “rbf”; alpha = 1.0; gamma = None
k-nearest neighbors	Extended	K Neighbors Regressor	n_neighbors = 5; weights = “uniform”; *p* = 2
Multilayer perceptron	Extended	MLP Regressor	hidden_layer_sizes = (100,); activation = ‘relu’; solver = ‘adam’; max_iter = 1000
Gaussian process	Extended	Gaussian Process Regressor	kernel = ConstantKernel(1.0) × RBF(1.0); normalize_y = True; alpha = 1 ×10^−10^
Logistic regression	Part A	LogisticRegression	penalty = ‘l2’; C = 1.0; solver = ‘lbfgs’; class_weight = ‘balanced’; max_iter = 1000
Random forest (classifier)	Part A	RandomForestClassifier	n_estimators = 300; min_samples_leaf = 5; class_weight = ‘balanced’

Global: all models were implemented in scikit-learn 1.9.0 with a fixed random seed of 42 for every data split and stochastic model; the hyperparameters listed above are those used, and any parameter not shown takes the scikit-learn 1.9.0 default.
